# Stalled replication forks within heterochromatin require ATRX for protection

**DOI:** 10.1038/cddis.2016.121

**Published:** 2016-05-12

**Authors:** M S Huh, D Ivanochko, L E Hashem, M Curtin, M Delorme, E Goodall, K Yan, D J Picketts

**Affiliations:** 1 Regenerative Medicine Program, Ottawa Hospital Research Institute, Ottawa, ON K1H 8L6, Canada; 2 Department of Biochemistry, Microbiology, and Immunology, Faculty of Medicine, University of Ottawa, Ottawa, ON K1H 8M5, Canada; 3 Department of Cellular and Molecular Medicine, Faculty of Medicine, University of Ottawa, Ottawa, ON K1H 8M5, Canada

## Abstract

Expansive growth of neural progenitor cells (NPCs) is a prerequisite to the temporal waves of neuronal differentiation that generate the six-layered neocortex, while also placing a heavy burden on proteins that regulate chromatin packaging and genome integrity. This problem is further reflected by the growing number of developmental disorders caused by mutations in chromatin regulators. ATRX gene mutations cause a severe intellectual disability disorder (*α*-thalassemia mental retardation X-linked (ATRX) syndrome; OMIM no. 301040), characterized by microcephaly, urogenital abnormalities and *α*-thalassemia. Although the ATRX protein is required for the maintenance of repetitive DNA within heterochromatin, how this translates to disease pathogenesis remain poorly understood and was a focus of this study. We demonstrate that *Atrx*^*FoxG1Cre*^ forebrain-specific conditional knockout mice display poly(ADP-ribose) polymerase-1 (Parp-1) hyperactivation during neurogenesis and generate fewer late-born Cux1- and Brn2-positive neurons that accounts for the reduced cortical size. Moreover, DNA damage, induced Parp-1 and Atm activation is elevated in progenitor cells and contributes to their increased level of cell death. ATRX-null HeLa cells are similarly sensitive to hydroxyurea-induced replication stress, accumulate DNA damage and proliferate poorly. Impaired BRCA1-RAD51 colocalization and PARP-1 hyperactivation indicated that stalled replication forks are not efficiently protected. DNA fiber assays confirmed that MRE11 degradation of stalled replication forks was rampant in the absence of ATRX or DAXX. Indeed, fork degradation in ATRX-null cells could be attenuated by treatment with the MRE11 inhibitor mirin, or exacerbated by inhibiting PARP-1 activity. Taken together, these results suggest that ATRX is required to limit replication stress during cellular proliferation, whereas upregulation of PARP-1 activity functions as a compensatory mechanism to protect stalled forks, limiting genomic damage, and facilitating late-born neuron production.

Mutations in genes encoding epigenetic regulators are the cause of many neurodevelopmental disorders, thereby highlighting the importance of chromatin remodeling to progenitor cell growth, competency, cell fate, and differentiation capacity.^1^ In this regard, mutations in the human *ATRX* gene cause *α*-thalassemia mental retardation X-linked (ATRX; OMIM no. 301040) syndrome, a severe intellectual disability disorder commonly associated with urogenital abnormalities, facial dysmorphism, and *α*-thalassemia.^2, 3^

The *ATRX* gene encodes a 280 kDa protein with two chromatin-interaction domains, a C-terminal SNF2 helicase-like domain that provides DNA-dependent ATPase activity and an N-terminal ADD (ATRX-DNMT3-DNMT3L) domain that serves as a dual histone modification recognition module (H3K9me3/H3K4me0; H3K9me3/H3S10p) to target ATRX to heterochromatin.^4, 5, 6^ Moreover, ATRX interacts with DAXX to form a histone chaperone complex that loads histone H3.3 onto telomeres, imprinted genes, and endogenous retroviral elements, to establish and maintain a heterochromatin environment.^7, 8, 9, 10, 11^ Nonetheless, it remains unclear how these biochemical functions contribute to brain development.

Forebrain-specific inactivation of *Atrx* in mice results in enhanced apoptosis and cerebral hypocellularity,^12^ a phenotypic feature commonly observed in ATRX patients.^13^ Further characterization of proliferating cells lacking *Atrx* demonstrate that S-phase progression is delayed and accompanied with an activated DNA-damage response, fragile telomeres, and mitotic catastrophe that enhances cell death in rapidly expanding progenitors of the testis, skeletal muscle, and CNS.^12, 14, 15, 16^ Aberrant replication of heterochromatin was suggested by ChIP-Seq analysis as Atrx binding sites are enriched at simple repeats, including telomeres and other guanine-rich sequences with a propensity to form G4 quadruplexes.^17^ Moreover, it was proposed that disease pathogenesis could arise from an inability to prevent G4-quadruplex formation, which would impede replication and transcription.^18, 19^ Initial support for this model came from studies showing that Atrx interacts with the Mre11-Rad50-Nbs1 (MRN) complex and that Atrx-deficient cells have an increase in stalled replication forks.^15, 20^ Mechanisms that protect stalled replication forks are especially critical during mid-late S phase, because of the abundance of natural barriers present in heterochromatin.^21^

Here, we examined whether Atrx functions to protect stalled replication forks from collapse and subsequent DNA damage. Indeed, we observed that *Atrx*-deficient cells acquire DNA damage in the S phase, which persists and accumulates in a cell-cycle progressive manner. The replication stress is defined by reduced colocalization of BRCA1 with RAD51, indicating aberrant replication fork protection. The degradation of replication forks is mediated by Mre11, which leads to an increase in double-strand DNA (dsDNA) breaks, fork collapse, genomic instability, and cell death that reduces the progenitor cell pool. As a consequence of fork degradation, neural progenitors activate poly(ADP-ribose) polymerase-1 (Parp-1) to promote fork protection and cell survival, thereby limiting upper layer neuron loss. Indeed, PARP-1 inhibition further perturbed cell growth. Moreover, acute knockdown (KD) of Daxx resulted in a similar degradation of nascent DNA strands, suggesting that histone H3.3 loading facilitates replication fork protection.

## Results

### Increased DNA damage in neural progenitors compromises late-born neuron production

**Figure 1:**
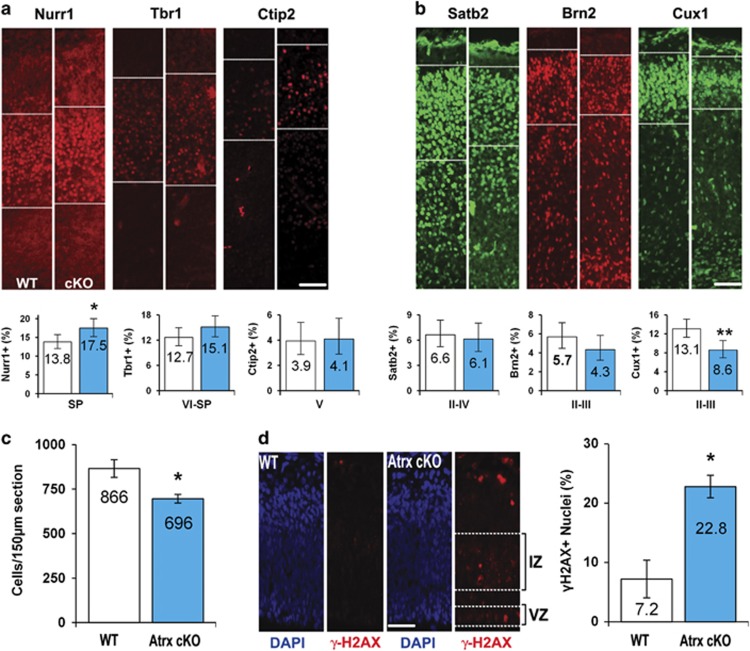
Atrx facilitates the production of late-born cortical neurons by preventing genomic instability in neural precursor cells. Representative micrographs and quantification of neurons located in the deep (**a**) or upper (**b**) neocortical layers from E18.5 *Atrx* cKO and WT coronal brain sections. Sections were probed with antibodies that specifically labeled the subplate (SP; Nurr1), layer VI-SP (Tbr1), and layer V (Ctip2), layers II–IV (Satb2), and layers II/III (Brn2 and Cux1). Labeled neurons within bounded areas were quantified as a percent of total nuclei within the neocortex. Values represent percent total±95% CI. *P<0.05 by *z*-score, whereas **P<0.01 by *z*-score; × 200 magnification. Scale bar, 100 *μ*m. (**c**) Average cell density counts from E18.5 WT and *Atrx* cKO cortical sections following DAPI staining. (**d**) Representative IF micrographs of E13.5 *Atrx* cKO and WT embryos coronal brain sections stained for *γ*-H2AX (red) or counterstained with DAPI (blue) to label all nuclei. NPCs reside in the VZ and IZ, as indicated by dotted lines; × 200 magnification. Scale bar, 100 *μ*m. Values represent proportional mean±S.E.M. *P<0.05 by Student's *t*-test

Previous work in our lab demonstrated that *Atrx*-null primary myoblasts were incapable of prolonged expansion owing to the S-phase defects and genomic instability that severely compromised muscle regeneration.^16^ If forebrain progenitor expansion was similarly affected, we reasoned that early-born neuron production would not be compromised but later born neuron production would be decreased, resulting in the reduced cortical mass we observed in *Atrx*^*FoxG1Cre*^ forebrain-specific conditional knockout (*Atrx* cKO) mice.^12^ To assess neuron production in *Atrx* cKO mice, we determined the proportion of cells comprising the different cortical layers using layer-specific markers. The earliest born neurons comprise the subplate and the deep layers (VI and V) of the cortex as the forebrain is generated in an inside-out manner. We observed a significant proportional increase in Nurr1+ subplate neurons but no differences in the layer VI (Tbr1+), layer V (Ctip2+), or layer IV (Foxp1+) cells in the *Atrx* cKO brains compared with wild-type (WT) littermates (Figure 1a and Supplementary Figure 1). While this suggested that a sufficient progenitor pool existed to generate the early-born neurons, we observed a significant reduction in the latest born Cux1+ neurons (layer II/III), whereas Brn2+ and Satb2+ neurons showed reduced levels that did not reach statistical significance (Figure 1b). Moreover, the cerebral cortex of *Atrx* cKO mice contained significantly fewer neurons than their WT littermates at E18.5 (Figure 1c), indicating that progenitor cell expansion was compromised.

**Figure 2:**
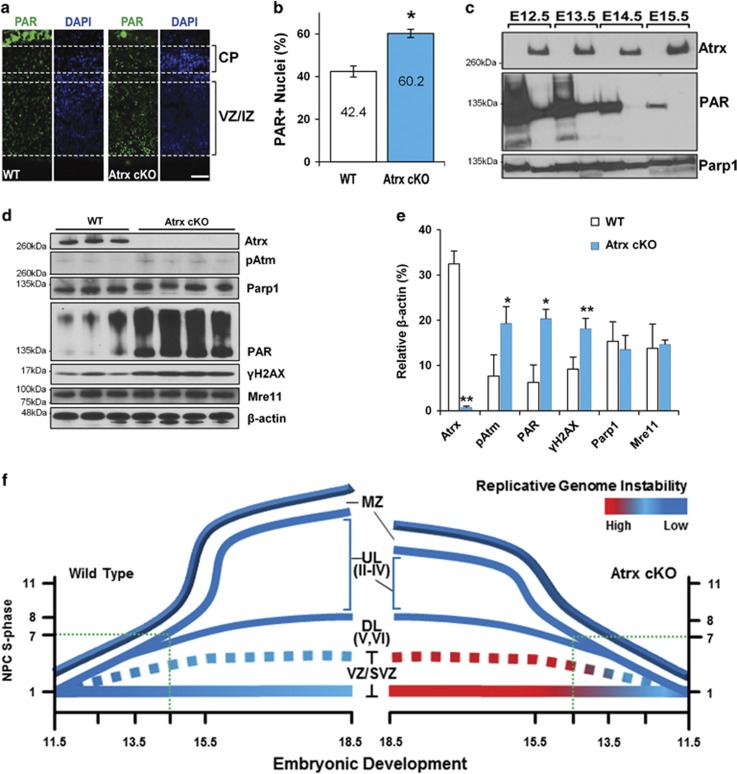
Enhanced activation of DNA-damage response pathways in *Atrx* cKO neuroprogenitors. (**a**) Representative IF micrographs of E13.5 coronal cortical sections from *Atrx* cKO and WT embryos stained with poly(ADP-ribose) antibodies (PAR; green) and counterstained with DAPI (blue). The cortical plate (CP) and NPC proliferative zones (VZ/IZ) are marked by dotted lines; × 200 magnification. Scale bar, 100 *μ*m. (**b**) Quantification of PAR-positive nuclei shown in (**a**). Values represent the mean±S.E.M.; *n*=3; *P<0.05 by Student's *t*-test. (**c**) Protein extracts from *Atrx* cKO and WT cortices were harvested daily from E12.5 until E15.5 and immunoblotted for Parp-1 activity (PAR), Parp-1 or Atrx. (**d**) Immunoblot analysis for DNA-damage signaling in E13.5 cortical extracts from WT (*n*=3) and *Atrx* cKO (*n*=4) embryos. (**e**) Densitometry quantification of blot shown in (**d**). Values are the mean±S.E.M. *P<0.05; **P<0.01, by Student's *t*-test. (**f**) Developmental model of replicative stress induced loss of late-born neurons in the *Atrx* cKO mice. The X axis shows the developmental time and the Y axis shows the number of cycles the NPCs have undergone. Blue lines depict the generation of deep (DL) and upper layer (UL) neurons. Dotted green lines indicate the timing of progenitor cell loss. At this point, progenitors from *Atrx* cKO mice within the VZ/SVZ (red line) have high levels of genomic damage that compromise their survival, resulting in a smaller cortex by E18.5

To determine whether genome instability might be the cause of reduced neuron production, we examined the DNA-damage marker *γ*H2AX by immunofluorescent (IF) staining of E13.5 cortical sections. We observed a significant increase in *γ*H2AX+ cells that was predominantly located in the proliferative ventricular (VZ) and intermediate (IZ) zones (Figure 1d). Furthermore, we observed an accumulation of genomic damage by E15.5 as assessed by the colocalization of *γ*H2AX signaling with markers for radial glial (Pax6+) and intermediate (Tbr2+) progenitor cells (Supplementary Figure 2). As the genomic instability in *Atrx* cKO myoblasts was caused by DNA replication stress, we examined Parp-1 activity, a known effector of this pathway. Parp-1 activity was assessed using antibodies specific to Parp-1 and polyADP-ribose (PAR), the moiety added to substrates when the polymerase is active. IF staining of E13.5 *Atrx* cKO neocortices revealed increased PAR staining primarily within the proliferative zone (Figures 2a and b). Immunoblots from cortical extracts demonstrated that this was not due to changes in Parp-1 expression but increased activity (Figure 2c). Indeed, a high level of PARylation was observed at E12.5 and E13.5 in all embryos but it persisted only in the *Atrx* cKO embryos at E14.5 and E15.5 (Figure 2c). As such, we used the E13.5 cortical extracts to assess the activation of the DNA-damage response via phosphorylation of ataxia telangiectasia-mutated (pATM) and H2AX (*γ*H2AX). Both mutant and WT samples showed active PARylation, but only *Atrx* cKO extracts showed increased pATM and *γ*H2AX to indicate an activated DNA-damage response (Figure 2c). Interestingly, the Parp-1 immunoblots show a shift in size only in the mutant lanes that probably reflects significant auto-PARylation of the Parp-1 protein (Figure 2c and Supplementary Figure 4). As an indication that DNA damage was leading to cell death, we harvested embryonic cortical extracts from *Atrx* cKO and WT littermates at E12.5 and E17.5 for caspase activity assays. We observed a significant increase in the activation of the executioner caspase, caspase-3, that was mediated by an intrinsic response, as we observed an increase in caspase-9 activity but not caspase-8 (Supplementary Figure 3).

Collectively, these data suggest that genomic instability within the neural precursor population contributes to the observed neuronal cell loss. As depicted in Figure 2d, we postulate that genomic damage accumulates with each successive pass through S phase in the *Atrx*-null progenitor cells, and with seven to eight cell cycles within the span of 3 days there is diminished viability, thereby reducing the pool of late-stage progenitors that generate the upper layer neurons.

### Delayed S phase in ATRX KD cells leads to increased activation of p53-ATM checkpoint in the subsequent G1

To further investigate the mechanisms by which ATRX regulates genomic stability, we generated both acute and stable KD HeLa cells using siATRX or short hairpin-expressing plasmids (psiRNA ATRX) with their respective controls (siScrambled (siScram) and psiRNA LacZ). Cell cycle progression analysis of BrdU-labeled cells revealed that psiRNA ATRX cells were delayed through S and G2–M phase, similar to primary myoblasts (Supplementary Figure 5; Huh *et al.*^16^). As extended passaging of our psiRNA ATRX-stable clones resulted in the selective suppression of the shRNA *ATRX* transgene, the remainder of our experiments used the acute KD model. Following transfection, protein levels of ATRX were nearly undetectable by 48 h and remained absent until 120 h, while we also observed an increase in *γ*H2AX signaling over this timeline (Supplementary Figure 6). As such, this model is able to replicate our *in vivo* results and can be used to explore the role of ATRX during replication stress.

**Figure 3:**
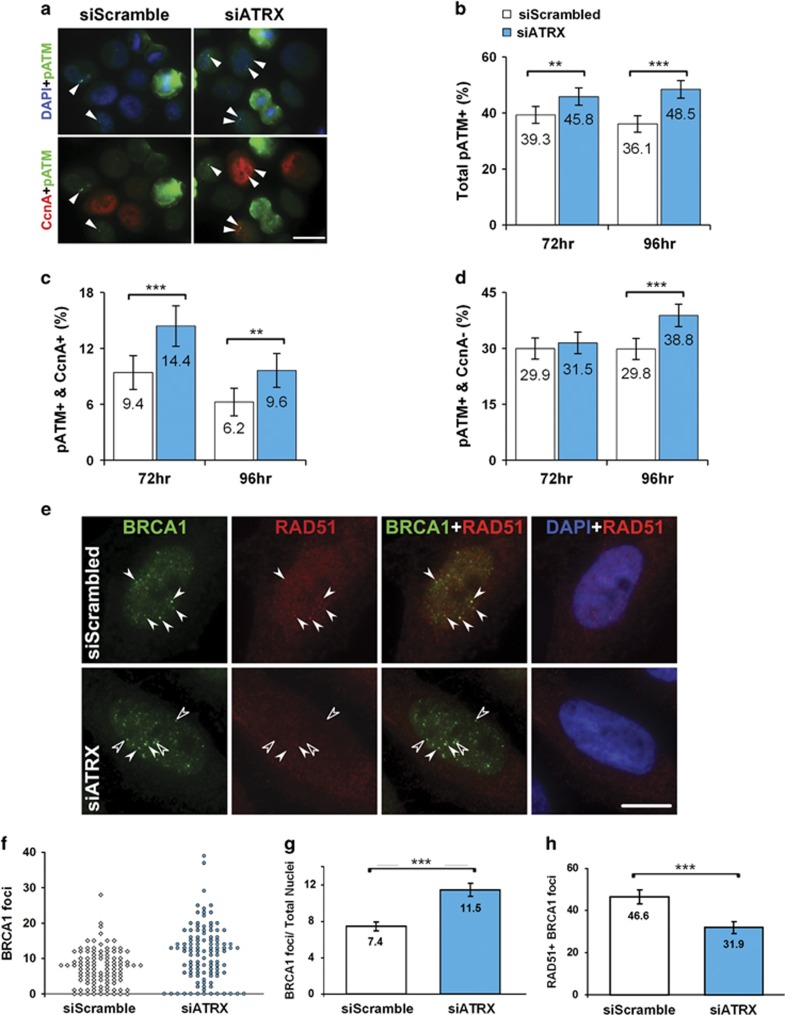
ATRX KD cells have increased activation of p53-ATM checkpoint upon mitotic progression and impaired RAD51 colocalization to BRCA1 foci. (**a**) Representative micrographs of phosphorylated ATM^Ser1981^ (pATM; green) and cyclin A (CcnA; red) double IF staining of siScram- and siATRX-transfected HeLa cells at 96 h post-transfection. Arrowheads point to cells with DNA-damage foci. (**b**) Percentage of total interphase nuclei containing pATM foci in siATRX- *versus* siScram-transfected HeLa cells at 72 and 96 h post-transfection. siATRX: 72 h, *n*=1001; 96 h, *n*=1007. siScram: 72 h, *n*=999; 96 h, *n*=1009. (**c**) Percentage of S-G2 (CcnA+) nuclei containing pATM foci at 72 and 96 h post-transfection. siATRX: 72 h, *n*=365; 96 h, *n*=366. siScram: 72 h, *n*=342; 96 h, *n*=308. (**d**) Percentage of G1 (CcnA−) nuclei containing pATM foci at 72 and 96 h post-transfection. siATRX: 72 h, *n*=636; 96 h, *n*=641. siScram: 72 h, *n*=657; 96 h, *n*=701. (**e**) Representative micrographs of BRCA1 and RAD51 double immunostaining in siScram control and siATRX KD HeLa nuclei 72 h post-transfection. Solid arrowheads point to foci that are BRCA1+ and RAD51+ and open arrowheads point to foci that are only BRCA1+. (**f**) Scatterplot distribution profile of BRCA1 foci from the experiment described in (**e**). siScram, *n*=106 nuclei; siATRX, *n*=111 nuclei. (**g**) Quantification of BRCA1 foci from the experiment described in (**e**). siScram, *n*=106 nuclei; siATRX, *n*=111 nuclei. (**h**) Percentage of total BRCA1 foci positive for RAD51 from the experiment described in (**e**). All images are at × 630 magnification; scale bars are 20 *μ*m (**a**) or 10 *μ*m (**e**). For graphs, values represent percent total±95% CI except for (**g**), which is mean the number of BRCA1 foci±S.E.M.; **P<0.01; ***P<0.001 by *z*-scores (**b**–**d** and **h**) or Student's *t*-test (**g**)

Previous work has demonstrated that Atrx-null cells are delayed through S phase and have an increased incidence of stalled replication forks.^15, 16, 20^ As stalled replication forks often collapse and form dsDNA breaks,^22^ we reasoned that the cell loss observed in ATRX KD cells may be due to the progressive accumulation of double strand breaks (DSBs) during progenitor proliferation. For this study, we examined the activation status of ATM with respect to cell-cycle stage (S/G2 or G1) at 72 and 96 h post-transfection. In this regard, cells were costained for pATM and cyclin A (Figure 3a). To quantify pATM signaling pertaining to DNA damage, cells with punctate staining were scored, while cytoplasmic pATM+ cells were excluded, as these represent cells undergoing mitosis.^23, 24^ Similarly, cells transiting S/G2 phases of the cell cycle were distinguished by cyclin A staining,^25, 26^ and this was confirmed in our hands (Supplementary Figure 7). At both the 72 and 96 h time points, we observed a significant increase in the proportion of ATRX KD cells (45.8% and 48.5%, respectively) with focal pATM nuclear staining compared with siScram (39.3% and 36.1%, respectively) control cells (Figure 3b). When total pATM cell counts were dissected into cells in S/G2 (cyclin A+) or G1 (cyclin A−) phase of the cell cycle, we observed a >50% increase in pATM staining in S/G2 phase at both 72 and 96 h (Figure 3c). Interestingly, we observed a time-dependent increase in pATM staining in the ATRX KD cells within the G1 sub-populations. The ATRX KD and control cells showed no difference at 72 h, but at 96 h post-transfection focal pATM staining significantly increased (compare 38.8% *versus* 29.8%) in the ATRX KD cells (Figure 3d). These findings illustrate the persistence and accumulation of a replication-dependent DDR response in the subsequent G1 of ATRX KD cells. Moreover, it further supports the model that progenitors accumulate more DSBs, ultimately resulting in genomic instability and activation of cell death pathways.

### Impaired RAD51 colocalization to BRCA1 foci in ATRX KD cells

Heterochromatin contains an abundance of simple repeats that are prone to instability during replication, forming unusual DNA structures (e.g. cruciform, Z-DNA, and G-quadruplexes) that can cause replication fork stalling.^21, 27^ ATRX is a heterochromatin-associated protein that preferentially binds to G-rich tandemly repeated DNA sequences that form G-quadruplexes.^17, 28^ As such structures require homology-directed recombination (HR) repair to remove them,^29^ we hypothesized that the absence of ATRX during replication may compromise the function of the HR machinery at replicating heterochromatin. In this regard, both ATRX and BRCA1 colocalized to replicating heterochromatin domains marked by either heterochromatin protein 1*α* (HP1*α*) or mid-late S-phase BrdU-labeled foci (Supplementary Figures 8A and B). To assess whether there was active HR repair after ATRX KD, we colabeled cells with BRCA1 and Rad51, functional beacons for HR machinery recruitment at sites of stalled replication forks.^22, 30, 31, 32^ Double IF detection of BRCA1 and RAD51 revealed colocalized nuclear focal signals (Figure 3e). Quantification of BRCA1 foci revealed a greater number of BRCA1 foci present in ATRX KD cells *versus* controls (compare 11.5 with 7.4 foci per nucleus respectively; Figure 3f). Despite this overall increase in the frequency of BRCA1 foci, the proportion of BRCA1 foci with colocalized RAD51 signals were markedly reduced in ATRX KD cells by 31% relative to controls (Figure 3h). Taken together, these data suggest that insufficient loading of RAD51 at BRCA1 foci may compromise HR-mediated fork restart or stability in the absence of ATRX.

### PARP-1 activation functions as a compensatory protective response to stalled replication forks

**Figure 4:**
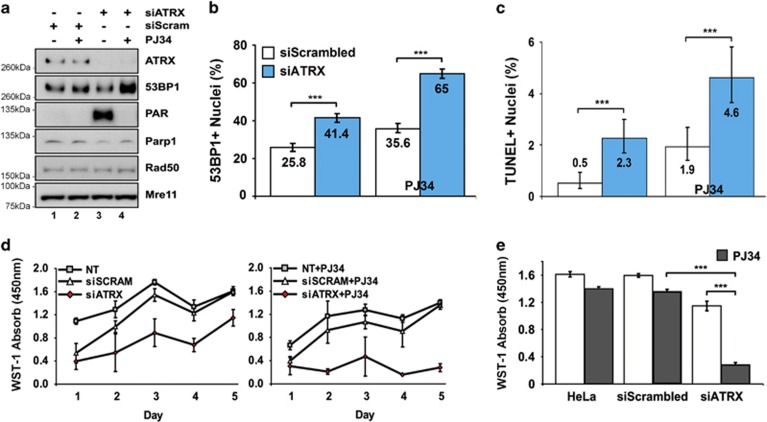
PARP-1 inhibition induces DNA breaks and causes growth suppression in ATRX KD cells. (**a**) Immunoblot analysis of PARP-1 inhibition by PJ34 in ATRX KD HeLa cells. As indicated, HeLa cells were transfected with siScram and siATRX. At 48 h after transfection, cells were treated with 5 *μ*M of PARP-1 inhibitor PJ34 (+) or untreated (−) for another 24 h. Whole-cell extracts were harvested 72 h post-transfection. (**b**) Percentage of total nuclei containing ⩾5 bright 53BP1 foci in siScram- *versus* siATRX-transfected HeLa cells at 96 h post-transfection. At 72 h after transfection, cells were treated with 5 *μ*M of PARP-1 inhibitor PJ34 (right) or untreated for another 24 h (left). Cells were fixed 96 h post-transfection and stained for 53BP1. Values represent percent total±95% CI. siScram (*n*=1420); siATRX (*n*=1607); siScram+PJ34 (*n*=1473); siATRX+PJ34 (*n*=1492). ***P<0.001 by *z*-scores. (**c**) Percentage of total nuclei containing TUNEL+ apoptotic nuclei in siScram- *versus* siATRX-transfected HeLa cells at 72 h post-transfection. At 48 h after transfection, cells were treated with 5 *μ*M of PARP-1 inhibitor PJ34 (right) or untreated for another 24 h (left). Cells were fixed 72 h post-transfection and TUNEL stained. Values represent percent total±95% CI. siScram (*n*=2251); siATRX (*n*=2031); siScram+PJ34 (*n*=1802); siATRX+PJ34 (*n*=1455). ***P<0.001 by *z*-scores. (**d**) WST-1 cell viability time course of untreated (NT), siScram-, siATRX-transfected HeLa cells. Cells were seeded equally 24 h following transfection (left panel) or treated with 5 *μ*M PJ34 24 h later (right panel). Viability measurements were assessed at day 1 (72 h post-transfection) until day 5. Values represent mean±S.E.M. For all conditions, *n*=4. (**e**) WST-1 cell viability measurement at day 5 of time courses described in (**d**)

We next questioned whether the increased PAR activity we observed in the *Atrx* cKO forebrain indicated a compensatory mechanism to protect stalled replication forks upon RAD51 dysregulation. PARPs are multifunctional enzymes that affect DNA repair, replication fork protection, and restart.^24, 33, 34, 35^ Moreover, PARP-1 hyperactivation in cells with compromised HR pathways has been attributed to a protective response induced by stalled and collapsed replication forks.^36, 37^ We first confirmed that increased PAR signaling was also detected in ATRX KD cells, while total PARP-1 levels remained unchanged (Figure 4a, compare lanes 3 and 1). In addition, we used siPARP-1 to attribute increased PARylation specifically to PARP-1. Indeed, PARP-1 accounts for ~90% of PARylation,^38^ and we observed a marked decrease in PAR signaling when cells were treated with both siATRX and siPARP-1 (Supplementary Figure 10). As other studies have shown that HR-deficient cells are commonly hypersensitive to PARP-1 inhibition,^39^ we used the PARP-1 inhibitor PJ34 to assess whether the ATRX KD cells were similarly sensitive. PARP-1 inhibition by PJ34 potently suppressed PAR signaling in ATRX KD cells, with a concomitant increase in 53BP1 protein levels compared with siScram controls (Figure 4a, lanes 4 and 3). Quantification of 53BP1-positive nuclei revealed an 83% increase in frequency within PJ34-treated ATRX KD cells relative to PJ34-treated controls (Figure 4b and Supplementary Figure 9A). Moreover, PJ34-treated ATRX KD cells showed an increased level of TUNEL+ (terminal uridine deoxynucleotidyl transferase dUTP nick-end labeling-positive) nuclei and a severe attenuation of their growth rate over a 5-day time course measured with a WST-1 cell viability assay (Figures 4c–e). Taken together, these experiments suggest that increased PARP-1 activity observed in the absence of ATRX represents a protective response to maintain the integrity of stalled replication forks.

### The ATRX-DAXX complex facilitates replication fork processivity and protection

**Figure 5:**
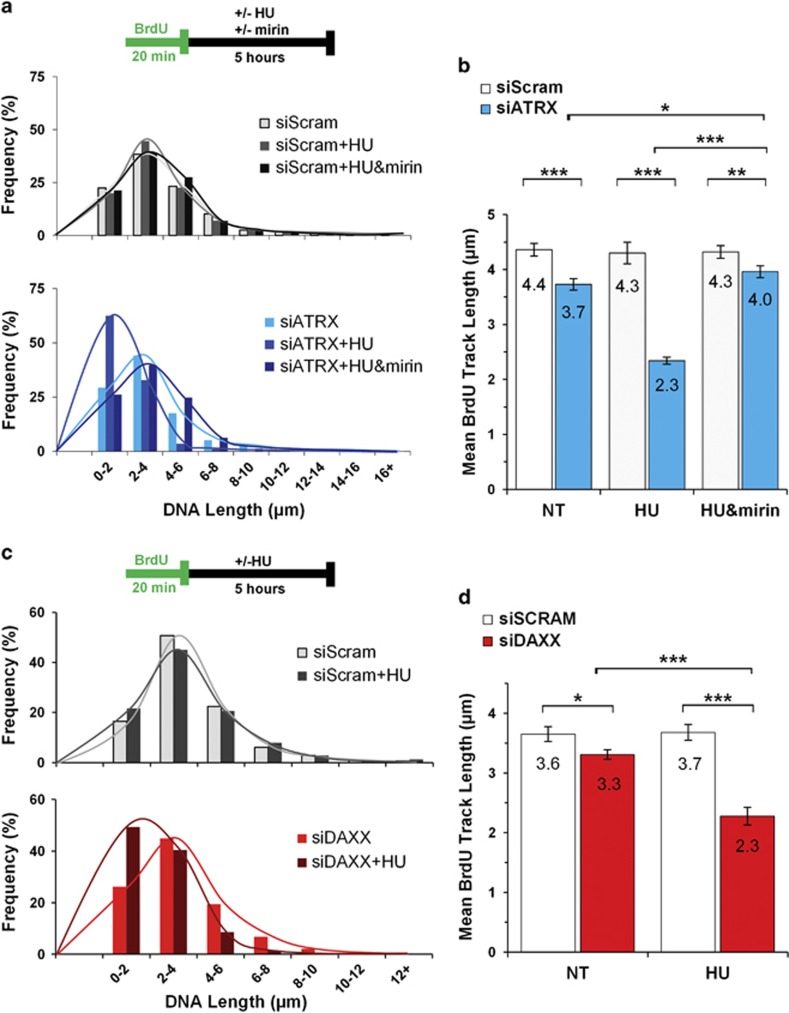
The ATRX-DAXX pathway protects stalled DNA replication forks from degradation by MRE11 exonuclease activity. (**a**) DNA fiber tract length distribution histogram of siScram- (top) and siATRX- (bottom) transfected HeLa cells at 72 h post-transfection. siRNA-treated cells were pulsed with BrdU and subsequently exposed to HU and mirin as indicated in the schematic. Total fibers counted for siScram experiment: no treatment, NT (*n*=1782); HU (*n*=1819); HU and mirin (*n*=1759). Total fibers counted for siATRX-treated cells: NT (*n*=1527); HU (*n*=1523); HU and mirin (*n*=1536). (**b**) Mean DNA fiber tract length of experiments described in (**a**). (**c**) DNA fiber tract length distribution histogram of siScram- (top) and siDAXX- (bottom) transfected HeLa cells at 72 h post-transfection. Fibers counted for siScram-treated cells were: NT (*n*=888); HU (*n*=998). Total fibers counted for siDAXX-treated cells were: NT (*n*=888) and HU (*n*=1171). (**d**) Mean DNA fiber tract length of experiments described in (**c**). For panels (**b** and **d**), the mean length±95% CI was plotted. *P<0.05, **P<0.01 and ***P<0.001 by Mann–Whitney test

ATRX-depleted ES cells exhibit a greater sensitivity to hydroxyurea (HU)-induced replication fork stalling and delayed replication restart.^15, 20^ These studies also identified a physical interaction between ATRX and the MRN complex.^15, 20^ However, the mechanism causing the increased fork stalling was not determined. Based on reduced Rad51 colocalization with BRCA1 and active PARP-1, we reasoned that replication fork protection could be compromised. In this regard, HR proteins such as BRCA1/2, RAD51, and MRE11 are functionally critical for the protection of stalled replication forks, independent of their role in dsDNA repair.^40^ RAD51 nucleofilament formation at stalled replication forks prevents MRE11-dependent degradation of newly synthesized DNA to allow for the resumption of DNA synthesis.^41^ Indeed, artificially blocking RAD51 nucleofilament formation by overexpressing the RAD51 binding peptide BRC4 potently induced fork destabilization upon HU exposure.^30^ To assess whether MRE11 exonuclease activity was overly active, we performed DNA fiber studies following HU-induced replication fork stalling, with or without ATRX present. Previous work has implicated BRCA1 in the protection of stalled replication forks.^30^ Indeed, we confirmed that BrdU-labeled nascent replication tracts of BRCA1-deficient cells (siBRCA1) were markedlly shorter following HU treatment compared with controls (Supplementary Figure 11). Quite strikingly, nascent replication tracts in ATRX KD cells were equally as short as the tracts observed in BRCA1 KD cells (Supplementary Figure 11). Shorter BrdU-labeled nascent DNA tracts may be the result of decreased replisome processivity rates and/or the instability to protect nascent strands from degradation at sites of stalled forks. To delineate the contribution of these processivity mechanisms, DNA track lengths were compared between the ATRX KD cells and siScram control cells without HU-induced fork stalling. While we observed that ATRX KD cells produced significantly shorter tracks than siScram control cells, track length reduction was significantly exacerbated upon HU treatment, indicating that fork protection is also compromised (Figure 5a). Moreover, chemical inhibition of MRE11 with the small molecule mirin has been demonstrated to protect stalled replication forks from exonuclease resectioning.^35, 42^ Indeed, mirin treatment of ATRX KD cells produced mean replication tract lengths that were comparable to that of controls (Figure 5b), suggesting that ATRX mediates MRE11-dependent degradation at stalled replication forks. Accordingly, ATRX may directly suppress MRE11-dependent degradation at stalled forks as it co-immunoprecipitates with both MRE11 and NBS1 in WT asynchronous cells (Supplementary Figure 12A). Regardless, H3.3 has been shown to facilitate replication fork processivity during replication stress and the ATRX-DAXX complex serves as a chaperone for loading this histone variant.^43, 44^ To determine if replication fork protection may be mediated by ATRX-DAXX loading of histone H3.3, we performed a DNA fiber assay after depleting Daxx protein expression using a targeted small interfering RNA (siRNA) (siDAXX). DAXX depletion did not affect ATRX protein levels (Supplementary Figure 12C), but did have a significant effect on DNA tract length (Figures 5c and d). Pertaining to processivity, tracts from siDAXX-treated cells without HU were shorter than those from siScram control cells; however, as with the ATRX KD, HU-induced fork stalling resulted in significantly shorter labeled tracts. These findings are consistent with a role for both ATRX and DAXX in the regulation of both replication fork processivity and protection upon fork stalling.

## Discussion

**Figure 6:**
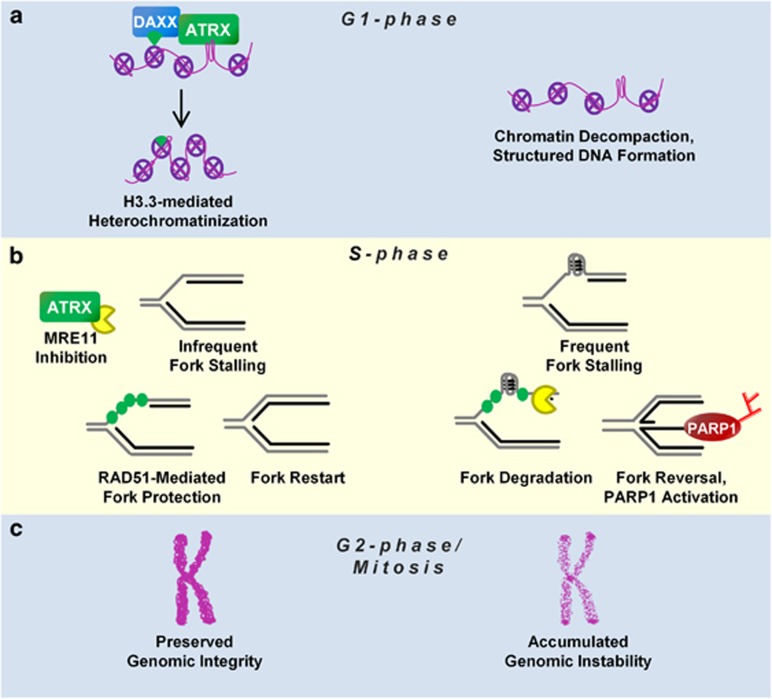
A model of how ATRX suppresses genomic instability during cellular proliferation. Relevant scenarios are shown during (**a**) G1 phase, (**b**) S phase and (C) G2/M phase in the presence (left) or absence (right) of ATRX. (**a**) During G1, ATRX localizes to decompacted and structured DNA (e.g. G4-DNA) along with DAXX to chaperone H3.3-H4 dimers that serve as a beacon for further heterochromatinization. When cells progress into the S phase (**b**), DNA replication forks experience more frequent stalling events when ATRX is absent owing to an increased incidence of structured DNA. ATRX physically interacts with MRE11 and inhibits excessive MRE11-mediated resectioning of stalled replication forks, which subsequently require RAD51-mediated protection of nascent DNA. In the absence of ATRX, PARP-1 activation is upregulated in an attempt to reverse stalled replication forks and protect against further MRE11 resectioning. Cells with frequent fork stalling that progress into the G2 phase and mitosis (**c**) are more prone to DSBs and mutagenic non-allelic homologous recombination events (NAHR) resulting in genomic instability

Neuronal progenitor cells of the ventricular (VZ)/subventricular (SVZ) zones sequentially exit the cell cycle to populate the distinct neuronal layers of the forebrain. Inherently, the most proliferative neural progenitor cells (NPCs) that become the upper neuronal layers have the greatest potential to incur replication-induced DNA damage and subsequent genomic instability. In this regard, we demonstrated that Atrx deletion *in vivo* in NPCs specifically compromised the genesis of cells targeted for the upper neocortical layers (Figures 1b and 2e). At the molecular level, we demonstrate that ATRX is required to diminish DNA replication stress, by protecting stalled replication forks, thereby preventing genomic damage and cell loss. Collectively, we propose a model in which ATRX is critical for heterochromatin maintenance throughout the cell cycle (Figure 6).

### ATRX and DAXX function to maintain heterochromatin stability

Simple repeats are poor substrates for nucleosome recycling during DNA replication and represent regions of latent epigenomic instability.^45, 46, 47, 48^ Heterochromatin environments are essential for the preservation of structural elements, such as centromeres and telomeres, as well as for the repression of malicious DNA sequences encoding endogenous retroviral elements. The ATRX-DAXX histone chaperone deposits H3.3 at globally diffuse heterochromatic loci, including telomeres, centromeres, differentially methylated regions, CpG islands, and endogenous retroviral elements in a replication-independent manner.^9, 11, 44, 49^ Accordingly, the loss of ATRX leads to the dysregulation of these loci^9, 16, 49^ and therefore we proposed a replication-independent mechanism for ATRX and DAXX to establish and maintain heterochromatin (Figure 6a). Although ATRX can recognize both HP1 and H3K9me3,^4, 50, 51^ its H3.3 chaperone function appears to be upstream of SUV39H-mediated H3K9 trimethylation.^9, 49^ Additionally, ATRX's ability to bind to G4 structured DNA *in vitro*, as well as its high binding enrichment at G4 motif containing DNA sequences *in vivo*,^17^ elicits the possibility that ATRX may recruit DAXX and H3.3 to G4 structured DNA for localized heterochromatinization (Figure 6a). Regardless, further experimentation is required to validate a role for ATRX in re-establishing heterochromatin, similar to studies identifying a role for Asf1 in histone recycling.^52^ Importantly, G4 structured DNA can cause replication fork stalling, necessitating its suppression before the S phase,^21^ whereas other studies have demonstrated fluid replication, although G4 motif DNA is required for the preservation of distinct epigenomic loci.^46, 47^

### ATRX actively protects stalled replication forks

Here we progress our model into the S phase and propose a mechanism wherein ATRX actively protects stalled replication forks within heterochromatin (Figure 6b). ATRX-deficient cells are burdened by increased replication fork stalling events,^15, 20^ which are subsequently degraded by MRE11 (Figures 5a and b) in a manner akin to BRCA1/2-deficient cells (Supplementary Figure 11).^30, 53^ Adapting a previous model for ATRX regarding telomere maintenance,^54^ we propose that ATRX physically sequesters MRE11 to inhibit its exonuclease activity, thereby preventing fork degradation. BRCA1 co-localization with RAD51 marks the protection of stalled replication forks,^29^ and we observed an increase in BRCA1 foci formation without a concomitant increase in RAD51 colocalization in ATRX-deficient cells. Unfettered MRE11 activity with an increased number of stalled replication forks may deplete RAD51 pools, and this may further attenuate stalled fork protection. In fact, a similar model has been proposed wherein ATR inhibition promoted precocious restart of stalled replication forks, thereby depleting RPA protein levels and ultimately leading to fork collapse.^55^ Alternatively, dysregulated heterochromatin proximal to G4 structrured DNA may cause ineffective mobilization of homologous recombination factors such as RAD51 in ATRX-deficient cells.

Furthermore, we propose that the upregulation of PARP-1 activity (Figures 2c and 4a) can be attributed to a compensatory mechanism that engages to protect stalled replication forks from MRE11-dependent degradation by PARP-1-mediated replication fork reversal^35, 56^ (Figure 6b). In this manner, the excessive processing of replicating heterochromatin in ATRX-null cells likely contributes to delayed S-phase progression (Supplementary Figure 5B; Clynes *et al.*^15^ and Huh *et al.*^16^). Therefore, unresolved replication intermediates become DSBs in the subsequent G2 phase,^57^ which may explain the increased DNA damage observed throughout the cell cycle (Figures 3b–d and 6c).

### Heterochromatin instability drives ATRX-associated disease

Collectively, our data and others' suggests that enhanced cell death and reduced tissue size occurs from an inability to faithfully replicate heterochromatin under periods of rampant proliferation. The replication intermediates lead to DSBs, genomic instability, and mitotic catastrophe that reduces cell number (Figure 6c). Paradoxically, ATRX loss in cancer is beneficial to cell survival through the promotion of the alternative lengthening of telomere (ALT) phenotype. In this regard, ATRX loss is believed to be a late event, presumably after sufficient growth control checkpoints are eliminated. The instability of telomeric heterochromatin in the absence of ATRX facilitates telomere sister chromatid exchange, which maintains telomere length in ALT. Conversely, reintroduction of ATRX into ATRX-null ALT cancer cells restores H3.3 deposition at telomeres, thereby inhibiting sister telomere exchange and causing growth suppression.^54^ Thus, our finding that small-molecule inhibition of PARP-1 activity attenuated growth of ATRX-deficient cells offers a potentially therapeutic avenue towards treatment of ALT-positive cancers, analogous to PARP-1 inhibitor treatment to eliminate BRCA1/2-deficient cancer cells.^58, 59, 60^

## Materials and Methods

### Animal husbandry

*Atrx* cKOs were generated by crossing ATRX floxed females (*ATRX*^*fl/fl*^) to ATRX^+/Y^:*FoxG1-Cre*^+/−^ males on a C57BL/6 background as described previously.^12^ *ATRX*^*fl/y*^*:FoxG1-Cre*^+/−^ and *ATRX*^*fl/y*^ (control) male littermates were harvested for analysis. Animal experiments were approved by the University of Ottawa's Animal Care ethics committee as per the guidelines set out by the Canadian Council on Animal Care.

### Generation of ATRX shRNA cell lines

The expression vector psiRNA-hH1neo (InvivoGen, Sand Diego, CA, USA) was digested with *Bbs*1 and purified for cloning the ATRX shRNA oligonucleotide. The ATRX sense (5′-ACCTAACACTCATCAGAAGAATCTGACCACCTCAGATTCTTCTGATGAGTGTTT-3′) and antisense (5′-CAAAAAACACTCATCAGAAGAATCTGAGGTGGTCAGATTCTTCTGATGAGTGTT-3′) oligonucleotides were designed with *Bbs*1 overhangs. The oligonucleotides (25 μM) were annealed in 150 mM NaCl by heating to 80 °C for 2 min followed by slow cooling to 37 °C. Annealed oligonucleotides were then ligated and cloned into the psiRNA-hH1neo plasmid. Recombinants were identified by an *Ase*I digestion, purified using a Qiagen Maxiprep Kit (Qiagen, Toronto, ON, Canada), and sent for sequencing (StemCore, OHRI, Ottawa, ON, Canada). To generate stable cell lines, HeLa cells (5 × 10^7^) were transfected with psiRNA expressing vectors by Lipofectamine (Life Technologies, Burlington, ON, Canada) as per the manufacturer's instructions. Clones were selected in DMEM supplemented with 800 *μ*g/ml G418 (Life Technologies) after 2 weeks in culture. Individual clones were isolated and KD of ATRX protein expression was determined by western blot.

### Cell culture

HeLa cells were cultured at 37 °C in DMEM with 10% fetal bovine serum (FBS) and 1% penicillin–streptomycin. Transient KD of ATRX and BRCA1 were performed on 50% confluent cells using 0.72% (v/v) INTERFERin (Polyplus, Illkirich, France) in Opti-MEM I Reduced Serum Medium (Thermo Fisher Scientific Inc., Waltham, MA, USA) as per the manufacturer's instructions, with 100 nM of either siATRX Smart Pool or a Scrambled control (GE Healthcare, Amersham, The Netherlands). siBRCA1 was a kind gift from Dr Christine Pratt (University of Ottawa, Ottawa, ON, Canada). PARP-1 was inhibited with 5 *μ*M PARP-1 inhibitor VIII (PJ34; Santa Cruz Biotechnology Inc.; sc-204161A).

For stable shRNA expressing clone growth curves, WT HeLa cells, psiRNA LacZ, and psiRNA ATRX-stable clones were G1 synchronized by 72 h serum withdrawal. Growth media were reintroduced at time 0 and cells were enumerated at the indicated time points.

### Protein extraction and immunoblot analysis

Cortical lysates were extracted by homogenization using the Tissue Tearor (Biospec Products Inc., Bartlesville, OK, USA) in RIPA buffer (1 × PBS, 1% NP-40, 0.1% SDS, 0.5% sodium deoxycholate, protease inhibitor Complete Mini EDTA-free in ddH_2_O). Cell culture lysates were extracted in RIPA buffer by gentle agitation. Protein samples were cleared by centrifugation at 4 °C and supernatants were quantified using the Bio-Rad Protein Assay reagent (Bio-Rad, Mississauga, ON, Canada). Protein samples were resolved on pre-cast 3–8% Tris-acetate or 4–12% Tris-Bis gels (NuPage; Life Technologies) and transferred onto PVDF membrane (Immobilon-P; Millipore, Etobicoke, ON, Canada). Membranes were probed with the indicated primary antibodies (see Supplementary Table S1) and HRP-conjugated secondary antibodies. Immunoblots were incubated with enhanced chemiluminescent substrate and signals were exposed to film. Densitometric gel analysis was performed using ImageJ (version 1.46r; Bethesda, MA, USA) software by integrating pixel density plots with background subtraction.

### IF microscopy for cell culture

Cells were grown on coverslips or cytospun (Cytospin 4 Cytocentrifuge; Thermo Fisher Scientific Inc.) onto slides and fixed in 2% PFA and permeabilized with 0.1% Triton-X. Primary antibodies (see Supplementary Table S1) were diluted in blocking buffer (20% horse serum, 0.1% FBS, 0.03% sodium azide, in PBS) and incubated overnight at 4 °C in a humidifying chamber. Secondary antibodies (Alexas 488 and 594; Life Technologies) were applied and nuclei were counterstained with DAPI. Images were taken with an Axio Imager M1 microscope (Zeiss, Toronto, ON, Canada) and analyzed using ImageJ software. Positively stained cells were scored as indicated, relative to DAPI-stained nuclei.

### IF microscopy for brain sections

Embryos were harvested at the indicated gestational time points. Heads from embryos were fixed in 4% PFA overnight at 4 °C. The heads were washed in PBS, cryoprotected in a 30% sucrose/PBS solution overnight at 4 °C, embedded in a 1:1 solution of 30% sucrose and OCT Compound (Tissue-Tek), and flash frozen on liquid nitrogen. Embedded tissue were serially sectioned at 10 *μ*m (Leica 1850 cryostat) and mounted onto Superfrost Plus-coated slides (Thermo Fisher Scientific) and dried at room temperature for 2 h. Slides were fixed with 70% ethanol for 5 min at 4 °C (IHC) or 2% PFA 10 min at room temperature and then rehydrated in 1 × PBS for 5 min before staining. When probing for PAR, slides were incubated in 2 N HC1 for 20 min at 37 °C. Sections were permeabilized (0.1% Tween-20, 0.1M Tris-HCl (pH 8.8)) and incubated in blocking buffer (20% goat serum, 0.3% Triton-X in PBS). Primary antibodies (see Supplementary Table S1) were diluted in blocking buffer and applied onto sections. Sections were washed in PBS, incubated in secondary antibody solution, and counterstained with DAPI. Images were taken with an Axio Imager M1 microscope (Zeiss). Marker-positive cell counts were performed on multiple (*n*>3) 200 *μ*M brain sections from the dorsal cortex and plotted as a percentage of the total number of DAPI-positive cells.

### Cell cycle progression analysis

HeLa psiRNA LacZ and psiRNA ATRX cells were pulsed with 30 *μ*M BrdU containing media in triplicate for each time point (0, 6, 12, 16, 20, 24, and 28 h). A total of 10^6^ cells were fixed with 1 ml of 70% ethanol solution at −20 °C, overnight, resuspended in 0.1 N HCl+0.7% Triton-X on ice for 15 min, and washed with PBS. Cells were stained in 1:100 dilution the primary antibody anti-BrdU (BD Biosciences, San Jose, CA, USA) diluted in HBT (PBS, 0.05% FBS, 0.005% Tween-20), washed with HBT, and stained with FITC-conjugated secondary antibody anti-mouse diluted 1:20 in HBT for 30 min in the dark and precipitated for 7 min at 1500 r.p.m. Cells were resuspended in PI (propidium iodide) solution with RNAse A (50 *μ*g/ml PI, 40 *μ*g/ml RNase A) at 2000 cells per *μ*l and analyzed by flow cytometry using a Beckman Coulter FACS station (Brea, CA, USA). Cell cycle distribution of the cell population was analyzed with the FCS Express 2 software (DeNovo Software, Thornhill, ON, Canada) and the cell-cycle profile of each time point was analyzed with the ModFit software (Verity Software House, Topsham, ME, USA).

### Caspase assays

Cortical lysate protein was added to freshly prepared caspase activity buffer (25 mM HEPES, 10% sucrose, 1 mM EDTA, 0.1% CHAPS, 10 mM DTT in ddH_2_O) for a total volume of 199 *μ*l per well. The reaction was initiated by the addition of 1 *μ*l of 10 mM fluorescent substrate (caspase-3 substrate, Ac-DEVD-AMC (P411; Biomol, Hamburg, Germany), caspase-8 substrate, Ac-IETD-AMC (P432; Biomol, Hamburg, Germnay), caspase-9 substrate, Ac-LEHD.AMC (P444; Biomol, Hamburg, Germany)) to each well. A ThermoLabsystems Fluoroskan Ascent FL fluorometer using an excitation filter set to 380 nm and an emission filter set to 460 nm was used to read the absorbance of each well every 5 min over a 2 h period.

### DNA fiber assay

Nascent DNA of HeLa cells treated with siATRX, siBRCA1 or siScram was labeled with a 50 *μ*M BrdU pulse and replication forks we stalled with 4 mM HU. Where indicated, cells were treated with the MRE11 inhibitor mirin at a concentration of 50 *μ*M. A total of 10^6^ cells per 2 *μ*l were spotted onto glass slides and lysed with 7 *μ*l of fiber lysis solution (50 mM EDTA, 0.5% SDS and 200 mM Tris-HCl) for 5 min at RT. Slides were tilted 15° to horizontal to spread DNA across the length of the slide, and then air-dried and fixed in methanol/acetic acid (3:1). Slides were immersed in 2.5 N HCl for 80 min, washed in PBS, blocked in 5% BSA and stained with 1:500 mouse anti-BrdU primary antibody (BD Biosciences), followed by 1:4000 donkey anti-mouse IgG (H+L) with Alexa Fluor 488 conjugate secondary antibody (Thermo Fisher Scientific Inc.). Indicated numbers of labeled DNA fibers from three independent experiments per condition were imaged (Zeiss Axio Imager M1 microscope, Oberkochen, Germany) and analyzed using the ImageJ software (Bethesda, MD, USA).

### WST assay

WST-1 proliferation assay was performed as per the manufacturer's instructions (ab65473; Abcam, Cambridge, UK). HeLa cells were seeded at 1000 cells per well on a 96-well plate and absorbance measured at 450nm.

### TUNEL assay

Cells were fixed with 2% PFA and were permeabilized in 0.1% Triton-X/0.1% sodium citrate for 2 min on ice. The TUNEL labeling was performed using the *In Situ* Cell Death Detection Kit (Roche Applied Science, Mississauga, ON, Canada) according to the manufacturer's instructions.

### Statistical analysis

Statistical analysis was performed using Microsoft Excel statistical analysis package with means and s.e. calculated. Significance was determined by two-tailed *t*-tests of unequal variance (95 and 99% confidence intervals). Additionally, *P*-values for fiber assays were determined by Mann–Whitney test. All significant *P*-values were marked with asterisks, as follows: *P<0.05, **P<0.01, and ***P<0.001.

## Abbreviations

ADD: ATRX-DNMT3-DNMT3L
ATRX: *α*-thalassemia mental retardation X-linked
DSB: double strand break
dsDNA: double-stranded DNA
HP1*α*: heterochromatin protein 1*α*
HU: hydroxyurea
IF: immunofluorescent
IZ: intermediate zone
KD: knockdown
MRN: MRE11-RAD50-NBS1
NPC: neural progenitor cell
PAR: polyADP-ribose
PARP-1: Poly(ADP-Ribose) polymerase1
pATM: phosphorylated ataxia telangiectasia-mutated
shRNA: short hairpin ribonucleic acid
siRNA: small interfering RNA
SVZ: subventricular zone
VZ: ventricular zone
