# Supplementary material for: Stalled replication forks within heterochromatin require ATRX for protection
Source: Cell Death Dis. 2016 May 12;7(5):e2220–. doi: 10.1038/cddis.2016.121 (PMC4917659; doi:10.1038/cddis.2016.121)
Supplement: Supplementary Information [file cddis2016121x1.pdf]

Supplementary Information

Stalled replication forks within heterochromatin require ATRX for protection.

Michael S. Huh[1,#], Danton Ivanochko[1,2,#], Emile L. Hashem[1,3], Maureen Curtin[1,2], Marilyne Delorme[1,2], Emma Goodall[1,2], Keqin Yan[1], and David J. Picketts[1-3,*]

[1] Regenerative Medicine Program, Ottawa Hospital Research Institute, Ottawa, Ontario, K1H 8L6, Canada;
[2] Department of Biochemistry, Microbiology, and Immunology, Faculty of Medicine, University of Ottawa, Ontario, K1H 8M5, Canada
[3] Department of Cellular and Molecular Medicine, Faculty of Medicine, University of Ottawa, Ontario, K1H 8M5, Canada

[#] These authors made equal contributions to the manuscript.

[*] To whom correspondence should be addressed: Regenerative Medicine Program, Ottawa Hospital Research Institute, 501 Smyth Road, Ottawa, Ontario, K1H 8L6, Canada; E-Mail: dpicketts@ohri.ca; Tel.: +1-613-737-8989; Fax: +1-613-737-8803.

Enclosed:

Supplementary Figures 1 – 12

Supplementary Table

Author contributions

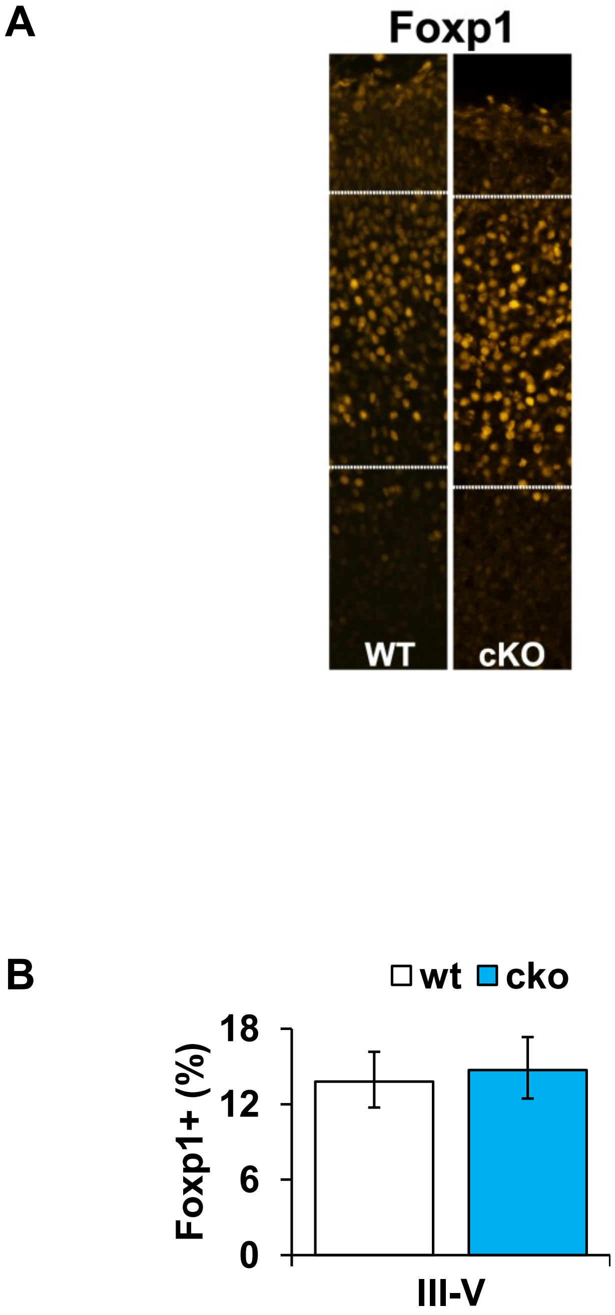

Supplemental Figure 1. WT and Atrx cKO mice have similar numbers of cells expressing the cortical layer IV marker Foxp1. Representative micrographs (A) and quantification (B) of Foxp1+ neurons within layer IV from E18.5 Atrx cKO and wildtype coronal brain sections. Labelled neurons were quantified as a percentage of total nuclei within the neocortex. Values represent percent total ± 95% CI. 200X magnification 200X. (Related to Figure 1.)

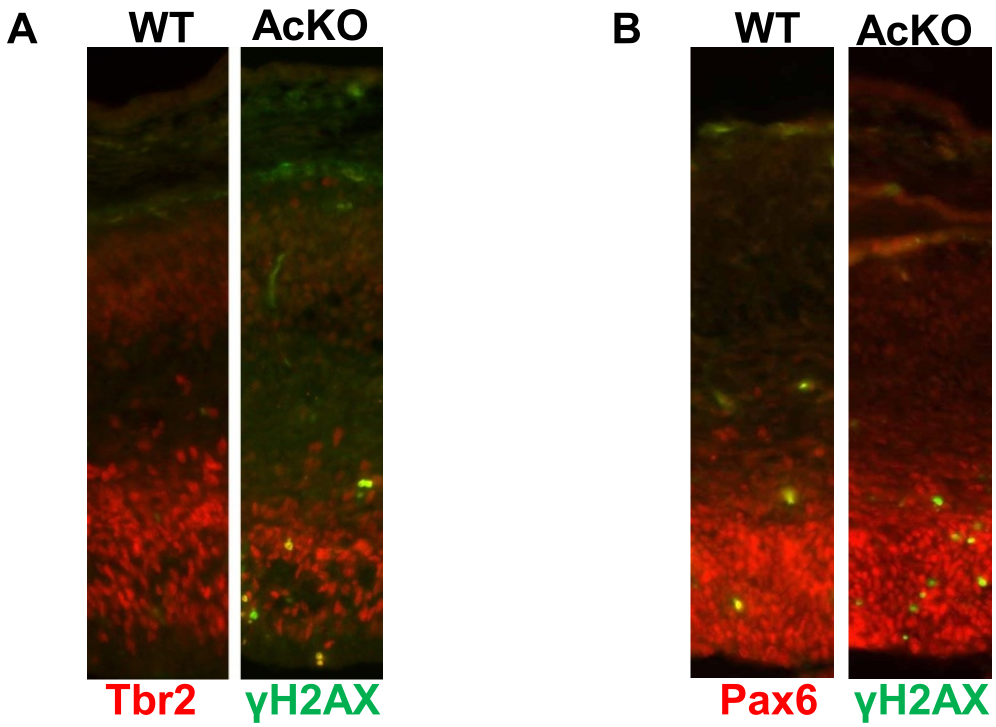

Supplemental Figure 2. Increased co-localization of γH2AX with Pax6 and Tbr2 in the forebrain of Atrx cKO mice. Representative micrographs of neurons stained for γH2AX and (A) Tbr2 or (B) Pax6 from E15.5 Atrx cKO and wildtype coronal brain sections. 200X magnification. (Related to Figure 1.)

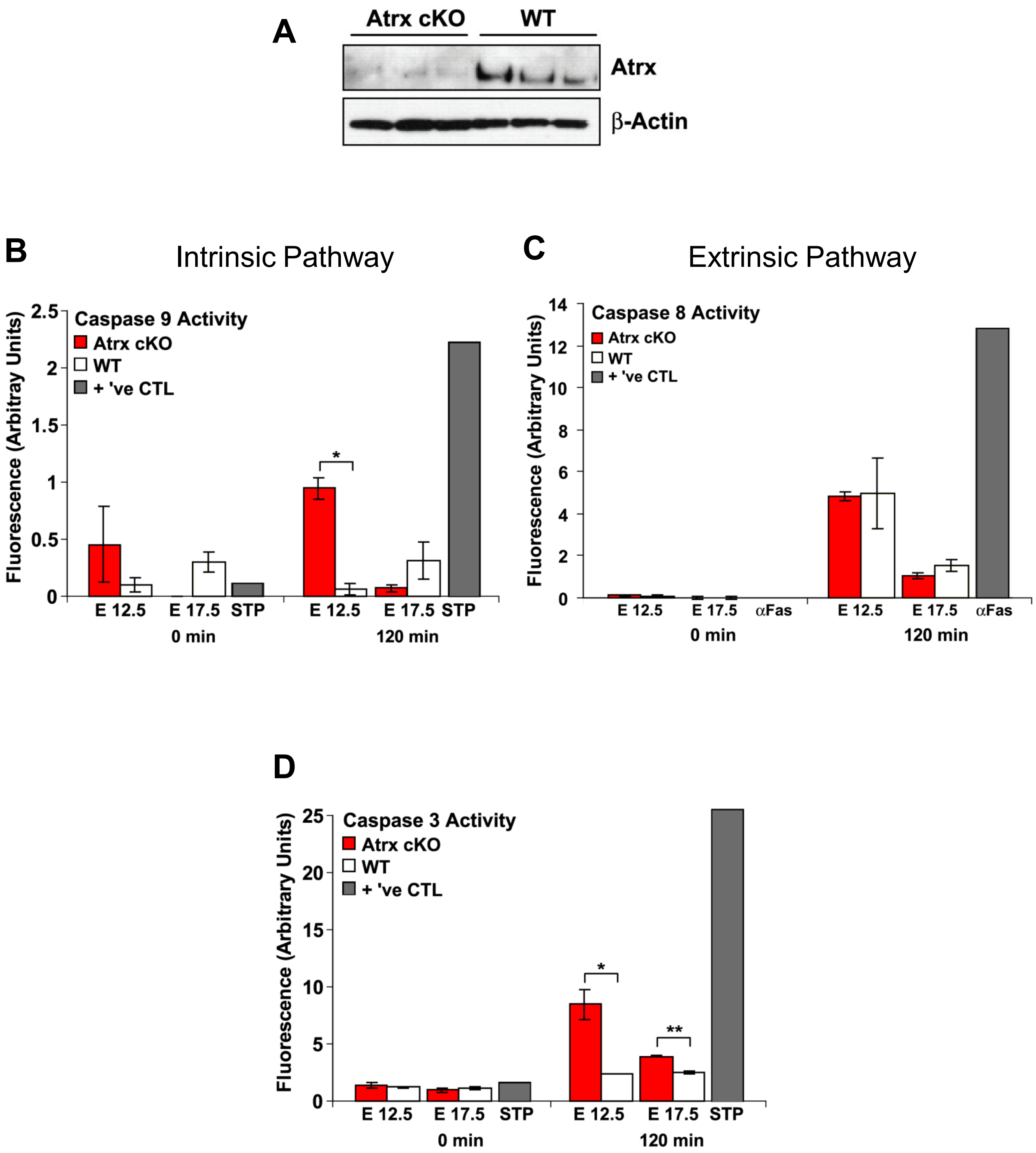

Supplemental Figure 3. Caspase activity assays from cortical extracts of WT and *Atrx* cKO embryonic brain. (A) Western analysis confirming Atrx loss from cortical extracts isolated from E12.5 WT and *Atrx* cKO mice. Enzymatic fluorometric assay for Caspase 9 (B), Caspase 8 (C) and Caspase 3 (D) activity in Atrx cKO and WT cortices at E12.5 and 17.5. Protein extracts from staurosporine (STP) treated N1E115 cells were used as positive controls for caspase activity. Values represent mean ± SEM. (*) $p < 0.01$, (**) $p < 0.001$ by student t-test. (Related to Figure 2.)

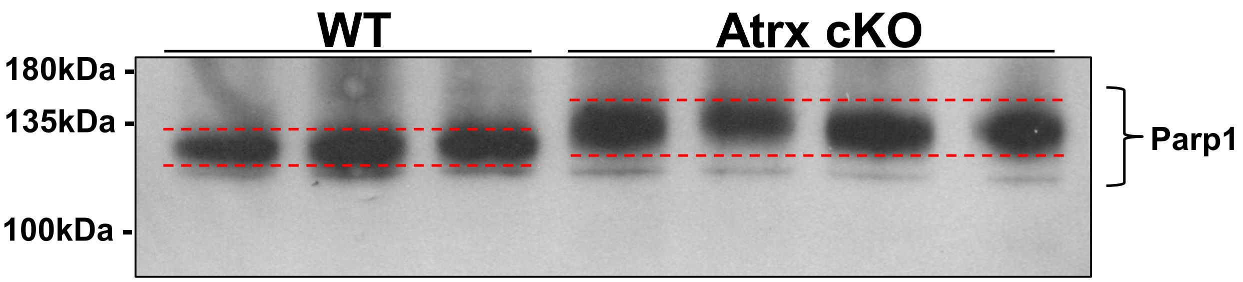

Supplemental Figure 4. Extracts from Atrx cKO brains display a shift in Parp1 migration. Mouse Parp1 (113kDa) was resolved on a 4-12% gradient gel from E13.5 wild type (n=3) and Atrx cKO (n=4) cortical extracts. The shift in Parp1 migration in the mutant lanes compared to WT lanes is indicative of auto PARylation or, alternatively, some other post-translational modification (Related to Figure 2.)

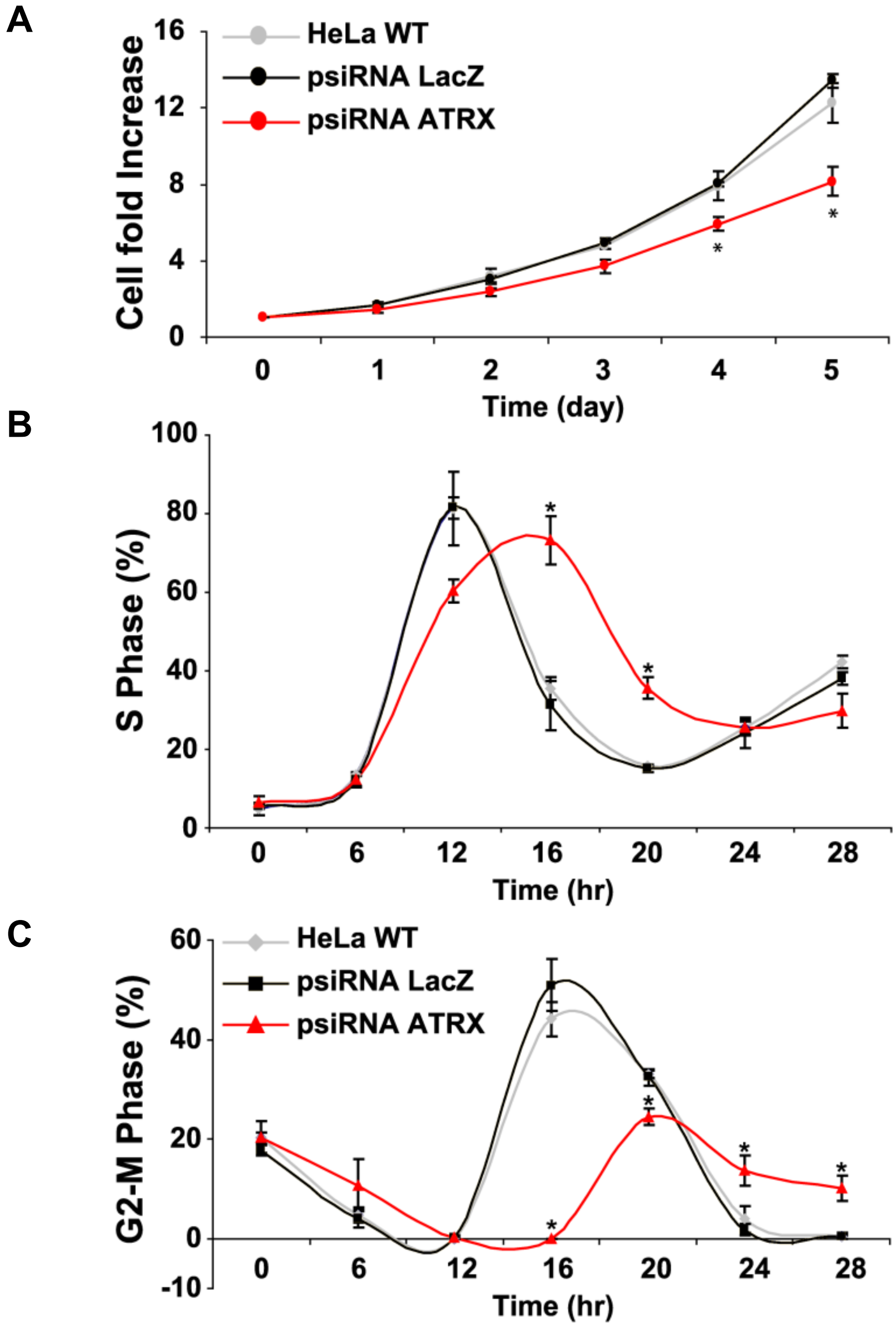

Supplemental Figure 5. ATRX KD cells display perturbed growth kinetics induced by delays in late-S phase. (A) Growth curve of stably selected ATRX knockdown shRNA expressing HeLa clones. Values represent mean fold increase ± SEM (n = 3 experiments per curve). (*) $p < 0.05$ by student t-test. (B - C) Flow cytometry cell cycle progression analysis of BrdU negative population from HeLa WT, psiRNA LacZ, and psiRNA ATRX expressing stable cell lines. Graphs represent percentage of all BrdU negative cells in the S-phase (B) and G2-M (C) gates at the indicated time points after the moment of BrdU removal (0 hr). Each data point represents proportion of cells ± SEM (n=4). (*) $p < 0.05$ by student t-test. (Related to Figure 3.)

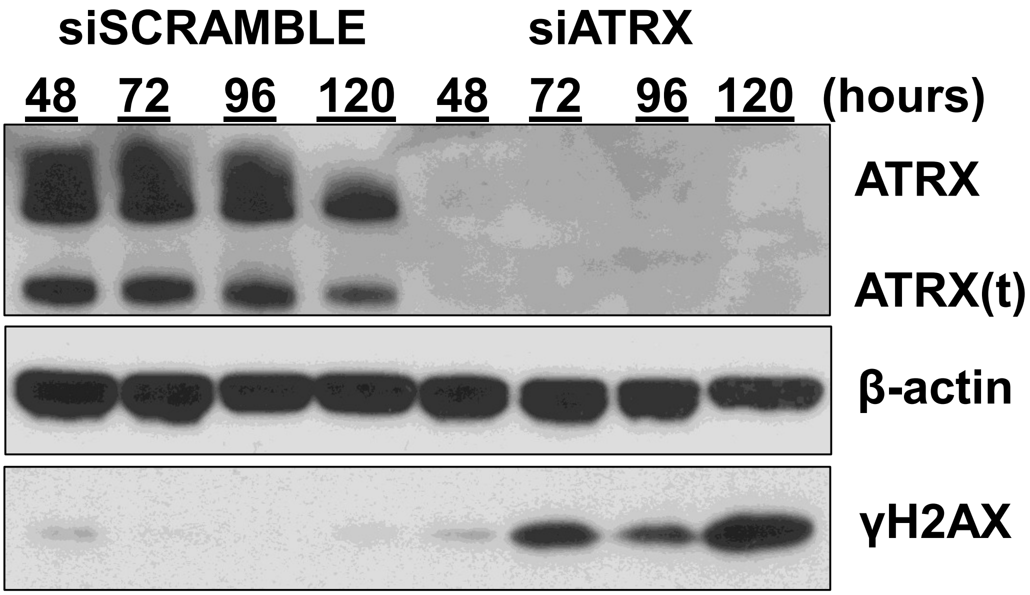

Supplemental Figure 6. ATRX expression in HeLa cells after siRNA treatment. Immunoblot analysis of siRNA mediated knockdown of ATRX examined at 48, 72, 96 and 120 hours post transfection relative to scramble siRNA control cells. A progressive increase of γH2AX expression was evident in KD cells. β-Actin serves as a loading control. (Related to Figure 3.)

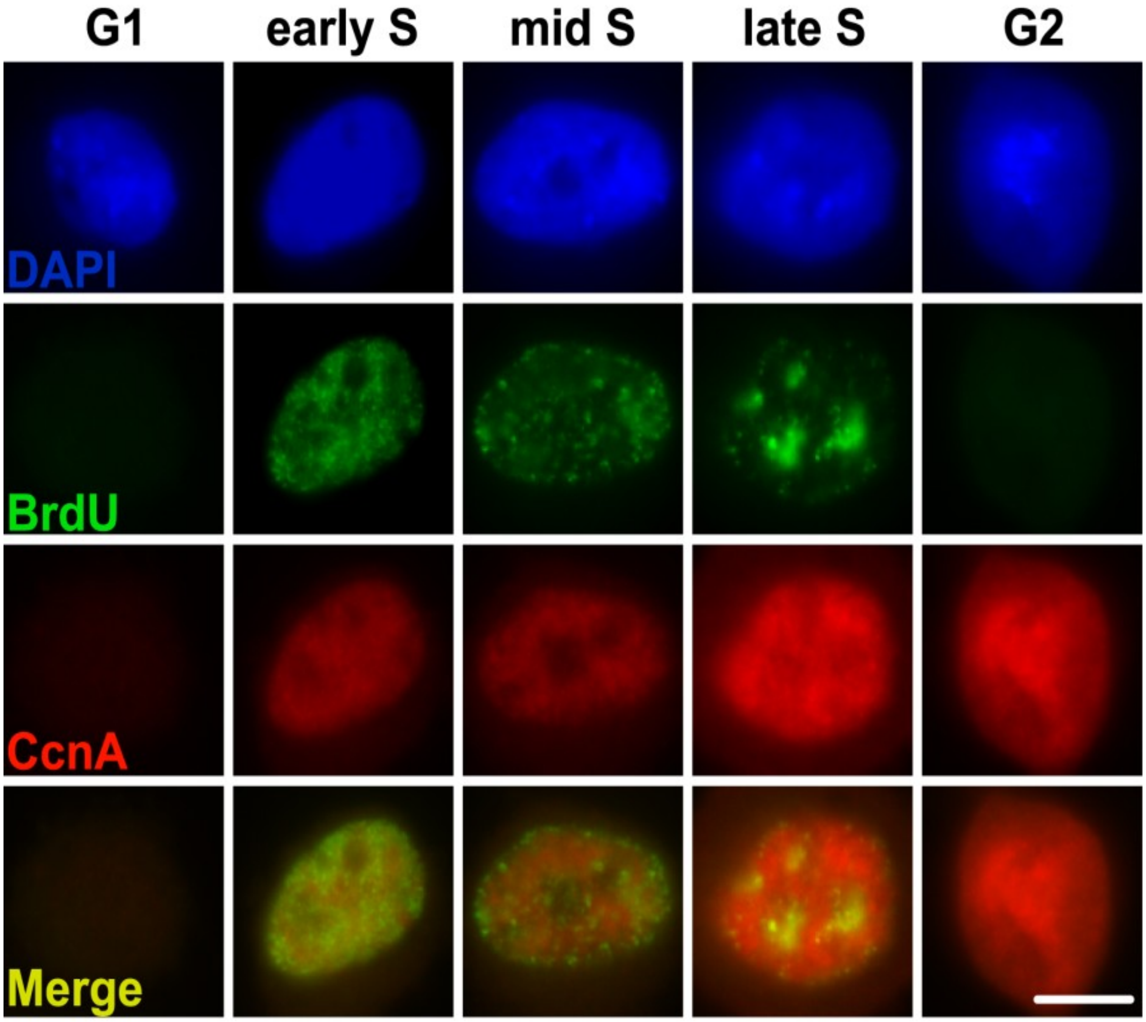

Supplemental Figure 7. Cyclin A is nuclear during S and G2 phases of the cell cycle. Representative micrographs of cell cycle stages in HeLa cells distinguished by double immunoflourescent staining for BrdU (green) and Cyclin A (red). Asynchronus HeLa cells were pulsed with BrdU for 20 minutes and immediately fixed in PFA for staining. 630X magnification. Scale bar, 10 µm. (Related to Figure 3.)

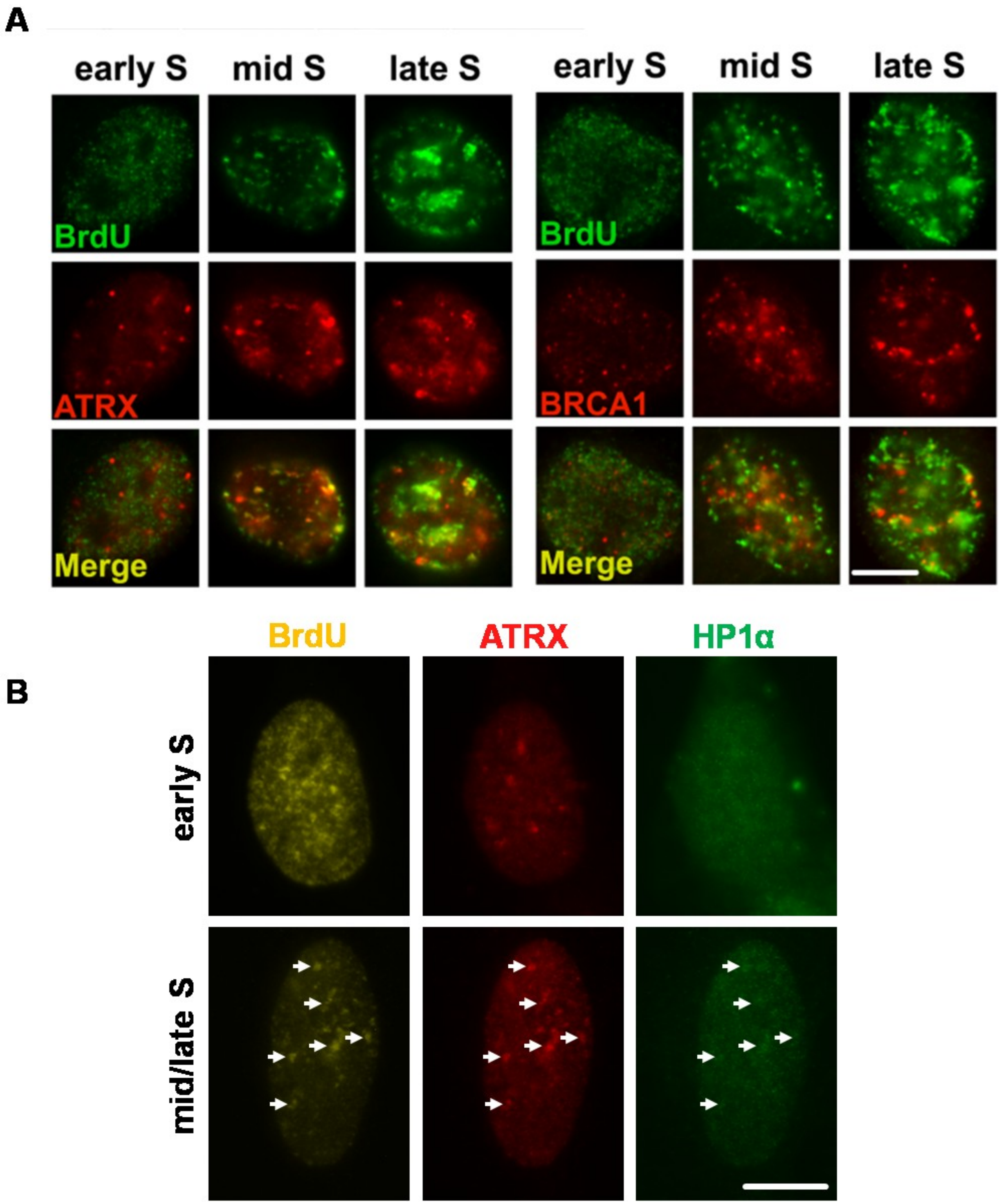

Supplemental Figure 8. ATRX localizes to late replicating heterochromatin. (A) S-phase specific double immunostaining for ATRX and BrdU. Wildtype HeLa cells were pulsed for 30 minutes with BrdU and immediately fixed. ATRX co-localized with mid-late replicating chromatin but not with early replicating chromatin (left panels). Similarly, BRCA1 foci were found adjacent and around the perimeter of mid-late replicating heterochromatin (right panels). 630X magnification. Scale bar, 10 µm. (B) Triple immunostaining for ATRX, heterochromatin protein 1 α (HP1α) and BrdU from unsynchronized WT HeLa cells. Fixed cells were stained with antibodies specific for ATRX (red) and HP1α (green) and BrdU (yellow). Colocalized foci of ATRX, HP1α and BrdU are indicated by arrowheads. Original magnification in (A –B) 400X. Scale bar, 10 µm. (Related to Figure 3.)

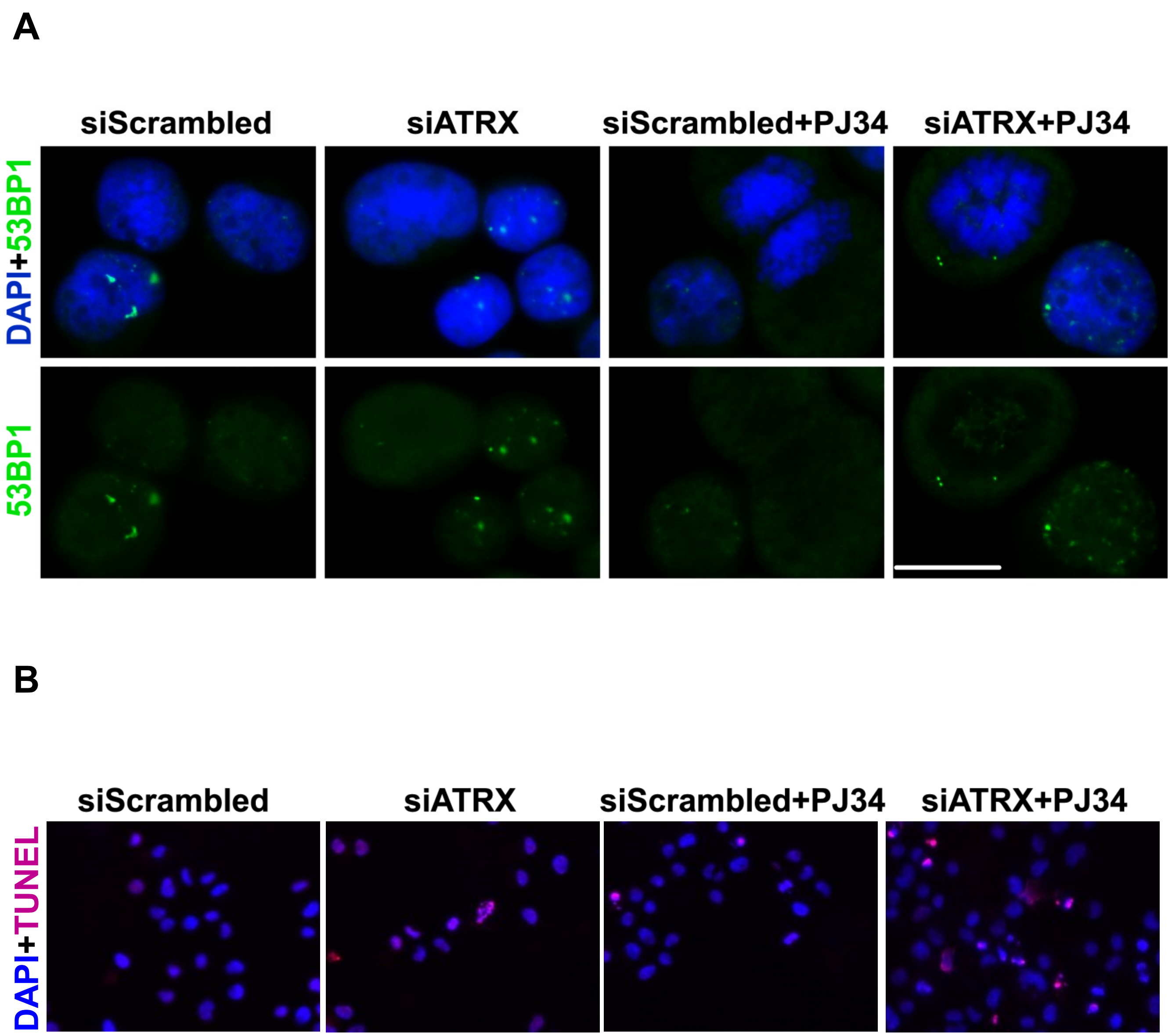

Supplemental Figure 9. PARP inhibition increases DNA damage foci in ATRX KD cells. (A) Representative immunofluorescent micrographs of 53BP1 staining in siATRX and siScrambled transfected HeLa cells. Cells were treated with the PARP inhibitor PJ34 (+PJ34) for 24 hours prior to fixation in PFA. 630X magnification. Scale bar, 20 µm. (B) Representative immunofluorescent micrographs of TUNEL staining in siATRX and siScrambled transfected HeLa cells. Cells were treated with the PARP inhibitor PJ34 (+PJ34) for 24 hours prior to fixation in PFA. (Related to Figure 4.)

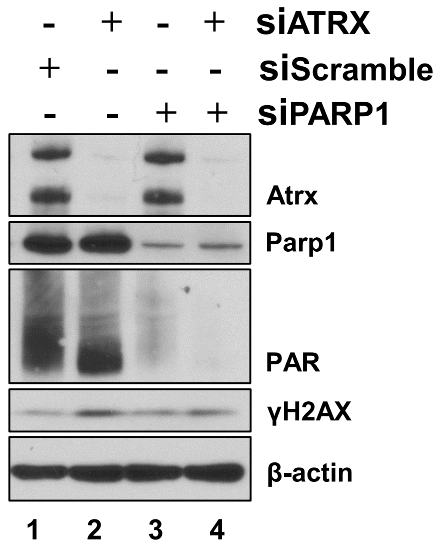

Supplemental Figure 10. PARP1 contributes to the PARylation induced in ATRX KD cells. Immunoblot analysis for PARP activation in HeLa cells following treatment with siATRX alone or combined with siPARP1. Whole cell protein lysates were harvested at 72 hours post-transfection for immunoblots of Atrx, Parp1, PAR, and γH2AX. β-actin serves as a loading control. (Related to Figure 4.)

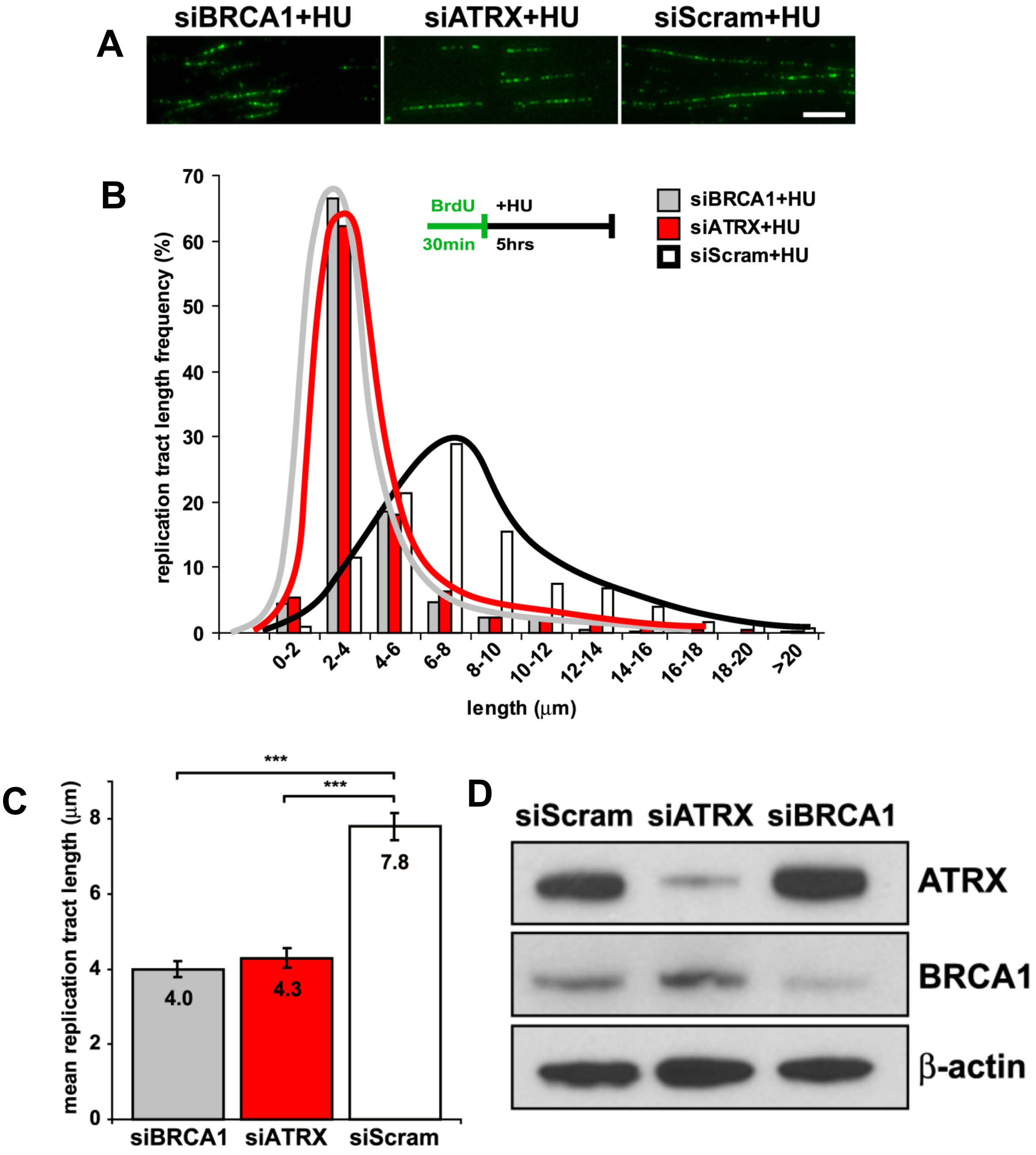

Supplemental Figure 11. Stalled replication forks are degraded in the absence of ATRX or BRCA1. (A) Representative images of BrdU-labelled DNA fiber tracts. 630X magnification. Scale bar, 2 µm. (B) DNA fiber tract length distribution histogram of siBRCA1, siATRX, and siScram control transfected HeLa cells at 72 hr post transfection. Insert shows schematic of experimental paradigm. HU, hydroxyurea. Number of DNA fibers measured: siBRCA1 (n = 514); siATRX (n = 528); siScram (n = 502). (C) Mean DNA fiber tract length of siBRCA1, siATRX and siScram control treated HeLa cells. Values represent mean length ± 95% CI. (***) $p < 0.001$ by Mann-Whitney test. (D) Immunoblots demonstrating knockdown of ATRX and BRCA1. β-actin served as the loading control. (Related to Figure 5.)

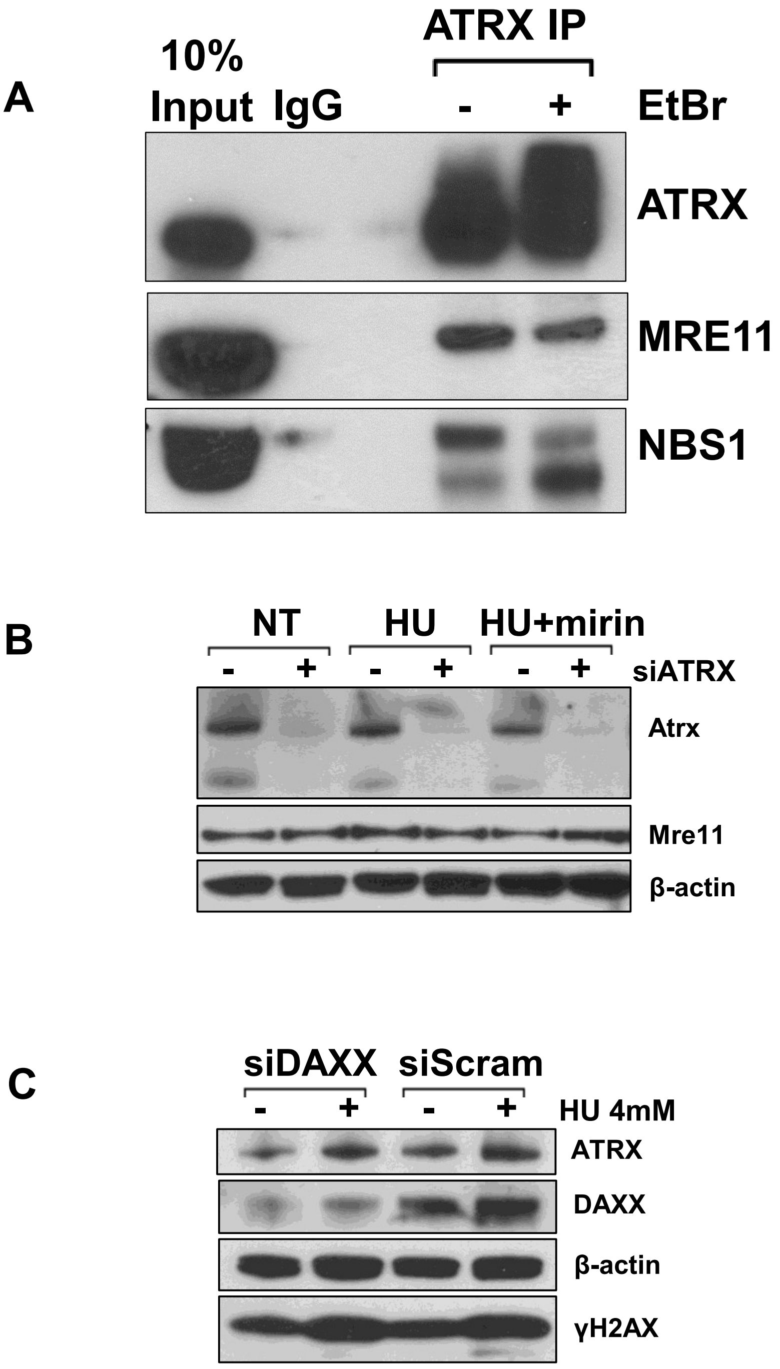

Supplemental Figure 12. Control immunoblots demonstrating ATRX-MRN interaction and protein KD after siATRX or siDAXX treatment. (A) Co-immunoprecipitation of MRN with ATRX from HeLa cell nuclear lysates. Immunoprecipitates were resolved by SDS PAGE and probed with antibodies specific for ATRX, as well as MRE11 and NBS1, two components of the MRN complex. Lysates were also treated with ethidium bromide (EtBr) to assess DNA dependent interactions. In, input; IgG, immunoprecipitates with rabbit IgG. (B) Representative western analysis demonstrating ATRX knockdown in HeLa cells after siATRX transfection alone (NT), or following hydroxyurea (HU), or HU and mirin treatments. Mre11 and β-actin immunoblots are shown for comparison. (C) Representative western analysis demonstrating DAXX knockdown in siDAXX but not siScram transfected cells, with (+) or without (-) HU treatment. (Related to Figure 5.)

Supplementary Table 1. Antibodies used in studies.

antibody	species	company/product no.	application (IF, WB, IP)
ATRX (F39)	mouse	Gift from D. Higgs	WB
ATRX (H-300)	rabbit	Santa Cruz/sc-15408	IF
ATRX (Fxnp5)	sheep	Custom manufactured	IP
β-actin	mouse	Sigma/A1978	WB
γ-H2A.X (20E3)	rabbit	Cell Signaling/9718S	WB, IF
p53	mouse	Clonetech/Ab6	WB
RPA32 (9H8)	mouse	Abcam/ab2175	WB
$ATM^{Ser1981}$	mouse	Millipore/MAB3806	IF
53BP1	rabbit	Novus/NB100-304	WB, IF
BRCA1 (D9)	mouse	Santa Cruz/sc-6954	IF
CyclinA (H-432)	rabbit	Santa Cruz/sc-751	IF
RAD51 (H-92)	rabbit	Santa Cruz/sc-8349	IF
PAR	rabbit	BD Pharmingen/ 551813	WB
PARP1	mouse	BD Pharmingen/556362	WB
MRE11	mouse	Abcam/ab214	WB
RAD50	rabbit	Abcam/ ab124682	WB
PAR	mouse	Trevigen/ 4335-MC-100	IF
BrdU	mouse	BD Pharmingen/347580	IF
Nurr1	rabbit	Santa Cruz/sc-990	IF
Tbr1	rabbit	Abcam/ab31940	IF
Ctip2	rat	Abcam/18465	IF
Foxp1	rabbit	Abcam/ab16645	IF
Satb2	mouse	Abcam/ab51502	IF
Brn2	rabbit	Santa Cruz/sc-28594	IF
Cux1	rabbit	Santa Cruz/sc-13024	IF

Author contributions

The manuscript was conceived and written by: M.S.H., D.I., and D.J.P.
In vitro experiments: M.S.H., D.I., E.H., M.D.
In vivo experiments: D.I., M.C., E.G., K.Y.
